# Nrf2-regulated redox signaling in brain endothelial cells adapted to physiological oxygen levels: Consequences for sulforaphane mediated protection against hypoxia-reoxygenation

**DOI:** 10.1016/j.redox.2020.101708

**Published:** 2020-09-08

**Authors:** Gabriela Warpsinski, Matthew J. Smith, Salil Srivastava, Thomas P. Keeley, Richard C.M. Siow, Paul A. Fraser, Giovanni E. Mann

**Affiliations:** King's British Heart Foundation Centre for Research Excellence, School of Cardiovascular Medicine & Sciences, Faculty of Life Sciences & Medicine, King's College London, 150 Stamford Street, London, SE1 9NH, UK

**Keywords:** Brain endothelial cells, Keap1-Nrf2, Heme oxygenase-1, Glutamate cysteine ligase, Ischemia-reoxygenation, Redox signaling, Sulforaphane, Oxygen, Physiological normoxia

## Abstract

Ischemic stroke is associated with a surge in reactive oxygen species generation during reperfusion. The narrow therapeutic window for the delivery of intravenous thrombolysis and endovascular thrombectomy limits therapeutic options for patients. Thus, understanding the mechanisms regulating neurovascular redox defenses are key for improved clinical translation. Our previous studies in a rodent model of ischemic stroke established that activation of Nrf2 defense enzymes by pretreatment with sulforaphane (SFN) affords protection against neurovascular and neurological deficits. We here further investigate SFN mediated protection in mouse brain microvascular endothelial cells (bEnd.3) adapted long-term (5 days) to hyperoxic (18 kPa) and normoxic (5 kPa) O_2_ levels. Using an O_2_-sensitive phosphorescent nanoparticle probe, we measured an intracellular O_2_ level of 3.4 ± 0.1 kPa in bEnd 3 cells cultured under 5 kPa O_2_. Induction of HO-1 and GCLM by SFN (2.5 μM) was significantly attenuated in cells adapted to 5 kPa O_2_, despite nuclear accumulation of Nrf2. To simulate ischemic stroke, bEnd.3 cells were adapted to 18 or 5 kPa O_2_ and subjected to hypoxia (1 kPa O_2_, 1 h) and reoxygenation. In cells adapted to 18 kPa O_2_, reoxygenation induced free radical generation was abrogated by PEG-SOD and significantly attenuated by pretreatment with SFN (2.5 μM). Silencing Nrf2 transcription abrogated HO-1 and NQO1 induction and led to a significant increase in reoxygenation induced free radical generation. Notably, reoxygenation induced oxidative stress, assayed using the luminescence probe L-012 and fluorescence probes MitoSOX™ Red and FeRhoNox™-1, was diminished in cells cultured under 5 kPa O_2_, indicating an altered redox phenotype in brain microvascular cells adapted to physiological normoxia. As redox and other intracellular signaling pathways are critically affected by O_2_, the development of antioxidant therapies targeting the Keap1-Nrf2 defense pathway in treatment of ischemia-reperfusion injury in stroke, coronary and renal disease will require *in vitro* studies conducted under well-defined O_2_ levels.

## Highlights

• Physiological normoxia alters the redox phenotype of murine microvascular brain endothelial cells.
• Intracellular GSH levels are lower in bEnd.3 cells adapted to 5 kPa versus 18 kPa O_2_.
• Nrf2 activated HO-1 and GCLM expression is attenuated under physiological normoxia.
• Sulforaphane protects against reoxygenation induced reactive oxygen species generation via Nrf2.

## Graphical abstract

**Figure:**
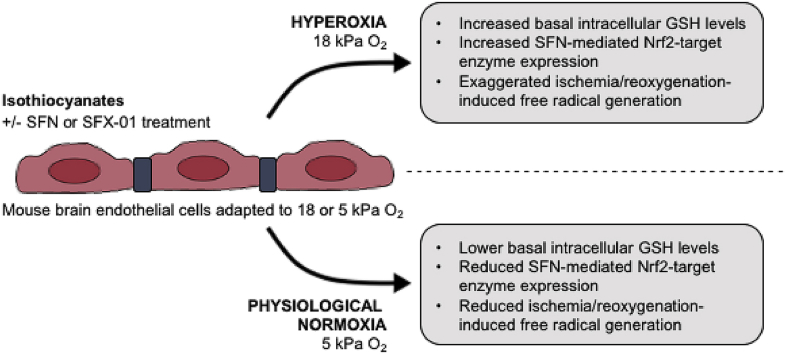

## 1 Introduction

Ischemic stroke is a leading cause of death and adult morbidity worldwide [1]. The critical reduction of blood flow within a major cerebral artery leads to reduced oxygen and nutrient delivery to the brain [2], time-dependent neuronal cell death and the development of neurological deficits [3]. The initiation of a pathophysiological cascade, involving oxidative stress and inflammation [4], is further exacerbated by the generation of reactive oxygen species (ROS) and mitochondrial dysfunction during reperfusion [5,6]. Timely restoration of cerebral blood flow is currently the only effective pharmacological treatment for acute ischemic stroke. Treatment with tissue plasminogen activator (rt-PA) improves reperfusion and functional outcomes, yet is limited to a ~4.5 h window after the onset of stroke due an increased risk of hemorrhagic transformation [7,8]. Recent trials of endovascular thrombectomy in stroke patients with large vessel occlusion report significant improvements in functional outcomes [1]. However, increased generation of reactive oxygen species in ischemic brain regions may compromise potentially rescuable penumbral tissue surrounding the infarct core [5,9].

Disruption of the blood-brain barrier (BBB) in ischemic stroke leads to extravasation of blood-borne inflammatory cells and fluid into the brain parenchyma which underlies dysregulation of neurovascular function [[10], [11], [12], [13]]. Our previous studies in a rodent model of ischemic stroke established that pretreatment of animals with the dietary isothiocyanate sulforaphane (SFN) [14], an electrophilic activator of the redox sensitive transcription factor Nuclear factor-erythroid 2 p45-related factor 2 (Nrf2) [15,16], significantly reduces BBB permeability, infarct volume and behavioral deficits [17,18]. Our MRI studies further demonstrated that prophylactic SFN delivery reduced lesion volume, consistent with reduced BBB permeability to IgG and improved neurological outcome [17]. Notably, SFN rapidly enters the brain [19] and upregulates Nrf2 and HO-1 expression in brain perivascular astrocytes and endothelial cells [17,18].

The majority of studies in endothelial and other cell types are conducted during culture under atmospheric O_2_ (18 kPa), whereas most cells experience much lower levels *in vivo*, with brain endothelial cells exposed to ~3–7 kPa [20]. Hyperoxic conditions create a pro-oxidation environment, reducing replicative lifespan [21] and enhancing cellular antioxidant defenses [22,23], thereby potentially limiting the clinical relevance of *in vitro* findings. We recently reported that SFN mediated induction of select Nrf2 target genes in umbilical vein endothelial cells (HUVEC) is attenuated under physiological normoxia (5 kPa O_2_) compared to atmospheric O_2_ levels [22]. Moreover, we reported that adaptation of HUVEC to 5 kPa O_2_ enhances nitric oxide bioavailability, modulates agonist-induced Ca^2+^ signaling [24] and protects against Ca^2+^ overload due to increased SERCA activity [25].

In this study, we further explore the mechanisms underlying SFN afforded protection in ischemic stroke by investigating redox signaling in mouse brain microvascular endothelial cells (bEnd.3) subjected to hypoxia-reoxygenation following adaptation to defined O_2_ levels. Our findings demonstrate that SFN induces Nrf2-regulated defense enzymes in bEnd.3 cells to protect against reoxygenation induced reactive oxygen species generation. These findings together with our study in of ischemic stroke *in vivo* [17,18] suggest that SFN may be a prophylactic therapeutic for targeting the Keap1-Nrf2 defense pathway in stroke and potentially coronary and renal disease.

## 2 Methods and materials

### 2.1 Culture and adaptation of bEnd.3 cells under defined O_2_ levels

Endothelialpolyoma middle T antigen transformed mouse brain microvascular endothelial cells (bEnd.3) were obtained from ATCC-LGC (Teddington, UK). Cells were cultured in phenol red free DMEM (Sigma, UK), supplemented with fetal calf serum (10%), l-glutamine (4 mM) and penicillin (100U/ml)/streptomycin (100 μg/ml). Cell monolayers were maintained for at least 5 days (d) in an O_2_-regulated dual workstation (Scitive, Baker-Ruskinn, USA), gassed to 18 kPa (hyperoxia), 5 kPa (physiological normoxia) or 1 kPa (hypoxia) O_2_ under 5% CO_2_ at 37 °C. This experimental protocol ensures adaptation of the cell proteome [20] and obviates re-exposure of cells to room air, as all cell culture, treatments and experiments are conducted within the O_2_-regulated workstation and/or plate reader (CLARIOstar, BMG Labtech, Germany). All experiments were conducted using bEnd.3 cells in passages 7–15.

### 2.2 Phosphorescence lifetime measurements of O_2_ levels in bEnd.3 cell cytosol and medium

Intracellular O_2_ levels were monitored in live cells using a cell-penetrating phosphorescent platinum–porphyrin based nanoparticle probe, MitoXpress®-INTRA (Agilent, USA) [26]. A time-resolved fluorescence plate reader (CLARIOstar, BMG Labtech), equipped with an atmospheric control unit, enabled us to measure cytosolic O_2_ levels under defined ambient O_2_ levels. bEnd.3 cells were seeded into 96-well black microtitre plates and loaded with MitoXpress®-INTRA (10 μg/ml) for 16 h in complete DMEM. The probe emits a phosphorescence signal at 655 ± 55 nm when excited at 355 ± 55 nm [22,24]. Molecular oxygen quenches the phosphorescence signal, and the signal decay is inversely proportional to the concentration of O_2_. Phosphoresence intensity after excitation was measured after 30 μs (*t*_1_) and 70 μs (*t*_2_) with a 30μs window and converted to probe lifetime (*τ*) using the formula: *τ*=(*t*_2_–*t*_1_)/ln (*f*_1_/*f*_2_), where f_1_ and f_2_ represent phosphorescence intensities at respective timepoints [27]. Averaged lifetime measured at 7 ambient O_2_ tensions was plotted against the known O_2_ tension and subjected to an exponential fit analysis. Lifetime values were then interpolated from this curve (see Fig. 2B) to give the dissolved intracellular O_2_ level in live bEnd.3 cells. Dissolved O_2_ culture medium was also measured in parallel by diluting MitoXpress®-INTRA (2.5 μg/ml) in DMEM medium.

**Fig. 1:**
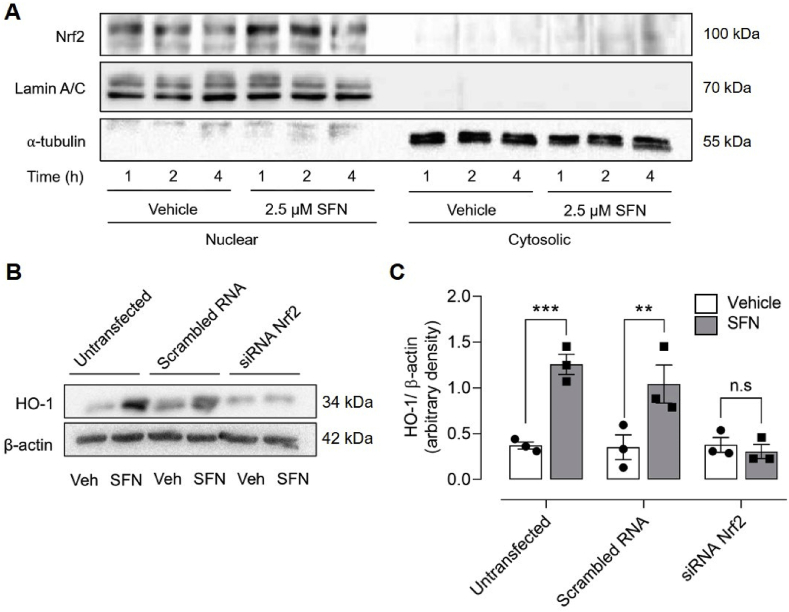
**Silencing Nrf2 abrogates induction of HO-1 by sulforaphane in bEnd.3 endothelial cells** (**A**) Representative immunoblot of Nrf2 in nuclear and cytosolic fractions isolated from bEnd.3 cells treated for 1, 2 and 4 h with vehicle (0.01% DMSO) or sulforaphane (SFN, 2.5 μM). Lamin A/C and α-tubulin are loading controls for nuclear and cytosolic fractions, respectively. (**B–C**) bEnd.3 cells were transfected with scrambled or Nrf2 siRNA 24 h post-seeding to silence Nrf2 transcriptional activity and then challenged with vehicle (0.01% DMSO) or SFN (2.5 μM) for 24 h. Cell lysates immunoblotted for HO-1 expression relative β-actin (**B**) and analysed by densitometry (**C**). Data denote mean ± S.E.M., n = 3 independent bEnd.3 cultures, two-way ANOVA, **P < 0.01, ***P < 0.001, n.s. non-significant.

**Fig. 2:**
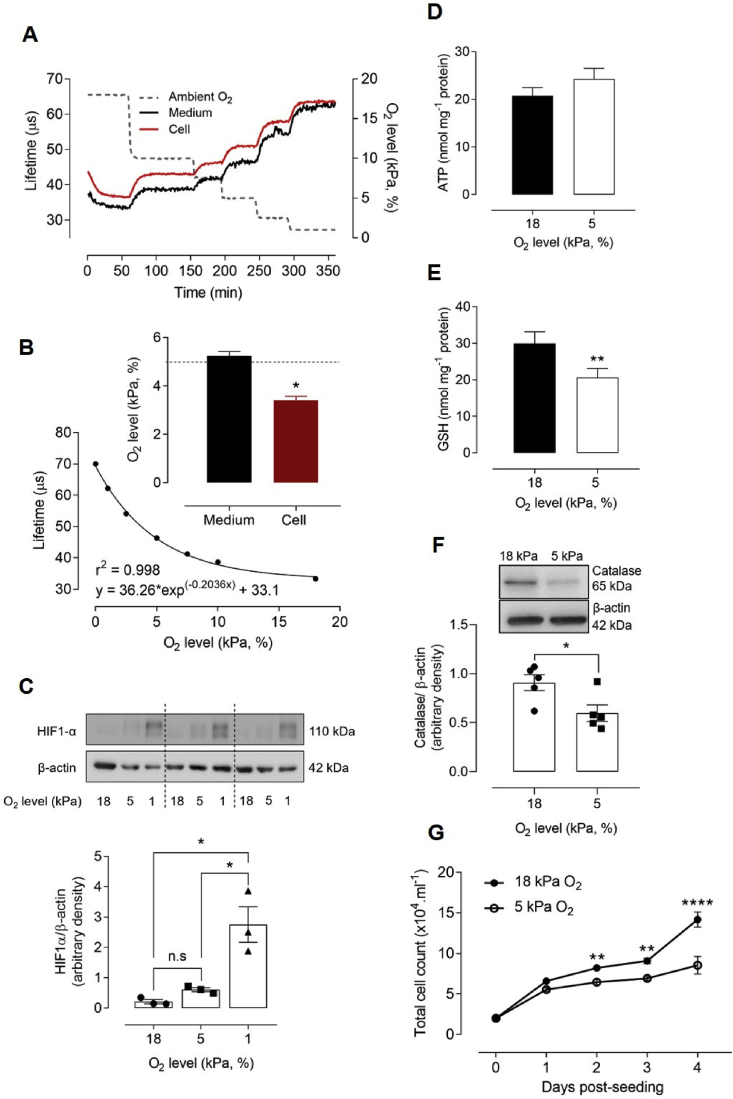
**Adaptation to 5 kPa O**_**2**_**alters the redox phenotype of bEnd.3 cells in the absence of HIF-1α stabilization** bEnd.3 cells adapted to 18 kPa O_2_ were loaded with MitoXpress®-INTRA for 16 h, transferred rapidly to an O_2_-regulated plate reader and exposed to stepwise reductions in O_2_ (dotted line, right axis). (**A**) Phosphorescence lifetime measurements (see Methods) in cells and dissolved O_2_ in medium. (**B**) Averaged phosphorescence lifetime *versus* ambient O_2_ levels in the plate reader were fit by exponential analysis. *Inset*: Interpolated O_2_ content in bEnd.3 cell cytosol and medium under 5 kPa O_2_ (dashed line). (**C**) Immunoblots of HIF-1α expression relative to β-actin and densitometric analysis of 3 cultures (separated by dashed lines). (**D–E**) Intracellular ATP and GSH levels in cells adapted for 5 d to 18 or 5 kPa O_2_. (**F**) Immunoblot and densitometric analysis of catalase expression relative to β-actin under 18 or 5 kPa O_2_. (**G**) Differential rate of bEnd.3 cell proliferation under 18 or 5 kPa O_2_. Data denote mean ± S.E.M., n = 3–5 independent bEnd.3 cell cultures, *P < 0.05, **P < 0.01, ****P < 0.0001, n.s. non-significant.

### 2.3 Immunoblotting

Cell lysates were extracted with SDS lysis buffer containing protease and phosphatase inhibitors (pH 6.8) on ice for 10 min. Denatured samples (10 μg) were separated by gel electrophoresis, electro-transferred onto polyvinylidene difluoride membranes and then probed with primary and HRP-conjugated secondary antibodies, using Lamin A/C (Santa Cruz, USA), α-tubulin (Millipore, UK) or β-actin (Sigma-Aldrich, USA) as reference proteins for nuclear and cell protein, respectively [22,28]. Nuclear protein was extracted using a nuclear extraction kit (Active Motif). Membranes were probed for HO-1 (Cell Signaling Technology), GCLM (gift from Prof. T. Kavanagh, University of Washington, WA, USA), NQO1 (Santa Cruz, USA), HIF-1α (Abcam, UK), catalase (Calbiochem, UK) and Nrf2 (Santa Cruz, USA). Protein expression was determined by enhanced chemiluminescence with images captured in a gel documentation system (G-BOX, Syngene Ingenius Bioimaging) and analysed by densitometry using Image J software (NIH, USA).

### 2.4 Quantitative RT-PCR

bEnd.3 cell RNA was isolated using a Nucleospin RNA Kit (Macherey-Nagel) and RNA content and purity assessed using a spectrophotometer (NanoDrop, Thermo Scientific, UK). Total RNA was reverse–transcribed using a high capacity cDNA conversion kit (Applied Biosystems) and HO-1, NQO1, GCLM, Bach1 and Keap1mRNA assessed by real-time qPCR (Corbett Rotorgene) [22,28] and normalized to the geometric mean of β-2-microglobulin (B2M), ribosomal protein L13a (RPL13A) and succinate dehydrogenase unit complex A (SDHA) (see primer sequences in Supplementary Table S1).

### 2.5 siRNA Nrf2 silencing

bEnd.3 cells were seeded at 30,000 cells/well and transfected with 40 pmol/well of either scrambled siRNA or Nrf2 siRNA (Santa Cruz, USA) [28] for 24 h using Dharmafect 4 transfection reagent (GE Healthcare, USA), as previously described [29].

### 2.6 Measurement of intracellular glutathione and ATP levels and cell viability

bEnd.3 cells were adapted to 18 or 5 kPa O_2_ for 5 days, and intracellular ATP and GSH extracted using 6.5% trichloroacetic acid (TCA, Sigma, UK). For ATP measurements, extracts were incubated with firefly lantern extract (Sigma, UK) containing both luciferase and luciferin, while total GSH levels were determined using a fluorometric assay [22,30]. Fluorescence and luminescence were measured in a plate reader (CLARIOstar, BMG Labtech). Cell viability was determined assaying mitochondrial dehydrogenase activity with 3-[4,5-dimethylthiazol-2-yl]2,5-diphenyl tetrazolium bromide (Sigma, UK) [29].

### 2.7 L-012 luminescence measurements of reactive oxygen species generation

bEnd.3 cells were seeded into white-walled, clear-bottomed 96-well plates and adapted for 5 d under 18 or 5 kPa O_2_. Confluent monolayers were incubated in low-serum medium (1% FCS) for 24 h prior to incubation in Krebs buffer in the absence or presence of superoxide dismutase (SOD, 100U/ml, Sigma, UK), polyethylene glycol SOD (PSOD, 50U/ml, Sigma, UK), polyethylene glycol catalase (PCAT, 200U/ml, Sigma, UK) or the NADPH oxidase inhibitor VAS2870 (VAS, 5 μM) [31] and the chemiluminescent luminol analogue L-012 (8-amino-5-chloro-7-phenyl-pyridol [3,4-d] pyridazine-1,4-(2H, 3H)dione sodium salt, 10 μM, Wako Chemicals) [32,33]. Cells adapted to either 18 or 5 kPa O_2_ were then rapidly (<30 s) transferred to an O_2_-regulated plate reader (CLARIOstar, BMG Labtech) at 37 °C and exposed to hypoxia (1 kPa O_2_) for 1 h and reoxygenation under 18 or 5 kPa O_2_, respectively. Luminescence was measured at 60 s intervals over 3 h and data expressed as mean light units/mg protein.

### 2.8 Mitochondrial reactive oxygen species measured using MitoSOX™ Red

Mitochondrial reactive oxygen species generation was measured using a mitochondrial targeted fluorogenic probe MitoSOX™ Red [34], and we previously confirmed that MitoSOX fluorescence in endothelial cells is attenuated by scavenging superoxide [28,35]. bEnd.3 cells seeded in black-walled, clear-bottomed 96-well plates were cultured under 18 or 5 kPa O_2_ for 5 d and then incubated in serum-free DMEM in the absence or presence of rotenone (1 μM, complex 1 inhibitor) or l-NAME (100 μM, eNOS inhibitor). Cells were exposed to hypoxia (1 kPa O_2_, 1 h) and loaded with MitoSOX™ Red (5 μM, Invitrogen) for 5 min before the start of reoxygenation under 18 or 5 kPa O_2_, respectively. Cells were washed twice with ice-cold PBS and fixed with 4% paraformaldehyde for 10 min before staining nuclei with DAPI (2 μg/ml, Sigma). MitoSOX™ Red fluorescence (Ex 545 nm/Em 602 nm) was detected using a Nikon Diaphot microscope, with images captured using an ORCA-03G (Hamamatsu, Japan) camera with 0.89 s exposure. Fluorescence quantification was conducted using image analysis software (ImageJ, NIH, USA), measuring the integrated intensity of fluorescence, area of field of view and mean grey value.

### 2.9 Intracellular free iron levels in bEnd.3 cells measured using FeRhoNox™-1

Intracellular iron release was measured using FeRhoNox™-1 (Goryo Chemical, Japan), a free iron turn-on fluorescent indicator specific for the detection of labile iron Fe(II) [36,37]. Cells in black, clear-bottomed 96-well plates were adapted to 18 or 5 kPa O_2_ and then incubated with FeRhoNox (5 μM) for 1 h, washed twice with PBS and incubated for 30 min with Hank's Balanced Saline Solution (HBSS, Gibco) containing either vehicle (DMSO, 0.01%), PEG-SOD (PSOD, 50U/ml) or the SOD inhibitor (ammonium tetrathiomolybdate, 4 μM) [38] before an assay. Fluorescence (Ex 540 nm/Em 575 nm) was measured in an O_2_-regulated plate reader (CLARIOstar, BMG Labtech).

### 2.10 Statistics

Data denote the mean ± S.E.M. of at least 3–5 different bEnd.3 cell cultures and were processed using Graphpad Prism 8, with some preliminary handling steps performed using MARS data analysis software (BMG Labtech). Significance was assessed using either a paired Student's *t*-test or one- or two-way ANOVA followed by a Bonferroni Post Hoc test where appropriate, with significance confirmed by P < 0.05.

## 3 Results

### 3.1 Sulforaphane induces Nrf2 nuclear translocation and antioxidant enzymes in bEnd.3 cells

Treatment of bEnd.3 cells under 18 kPa O_2_ with the Nrf2 inducer sulforaphane (SFN, 2.5 μM) increased nuclear accumulation of Nrf2 over 1–2 h (Fig. 1A). Nrf2 gene silencing had negligible effects on basal HO-1 protein levels but abrogated SFN-induced upregulation of HO-1 (Fig. 1B and C) and NQO1 (data not shown) expression. In initial experiments, we established that physiological concentrations of SFN (0.5–2.5 μM) [14] significantly upregulated Nrf2 mediated HO-1 protein levels (12–24 h, Supplementary Fig. S1A) and mRNA expression of HO-1, NQO1, Bach1 and Keap1 (4 h, Supplementary Fig. S1B).

### 3.2 Real-time measurement of intracellular O_2_ level in bEnd.3 cells

We and others have emphasized the importance of monitoring O_2_ gradients between culture medium and cell cytosol [20,22,39,40], and here report the first real-time measurement of intracellular O_2_ in brain microvascular endothelial cells (bEnd.3) using the cell-penetrating phosphorescent nanoparticle probe MitoXpress®-INTRA [26]. Phosphorescence lifetime in bEnd.3 cells and medium was measured during stepwise reductions of O_2_ (18 kPa–0 kPa) within an O_2_-regulated, time-resolved fluorescence plate reader (Fig. 2A). The relationship between ambient O_2_ levels in the plate reader and phosphorescence lifetime is illustrated in Fig. 2B. An intracellular O_2_ level of 3.4 ± 0.1 kPa (*inset*, Fig. 2B) was measured in cells cultured under 5 kPa O_2_, recapitulating levels in the cortex of awake mice [41], with dissolved O_2_ in the medium (5.2 ± 0.2 kPa) similar to the O_2_ level (5 kPa) in the plate reader.

### 3.3 Adaptation of bEnd.3 cells to 5 kPa O_2_ does not induce a hypoxic phenotype

To determine whether adaptation of bEnd.3 cells under 5 kPa O_2_ induces hypoxic responses, we examined stabilization of HIF-1α, a key modulator of transcriptional responses to hypoxia. In the presence of oxygen, HIF-1α is degraded via prolyl hydroxylation [42], involving HIF-1α association with von Hippel-Lindau protein E3 ubiquitin ligase complex to promote degradation [42,43]. As intracellular O_2_ availability decreases, these enzymes are no longer able to hydroxylate HIF-1α subunits, resulting in stabilization and upregulation of HIF-1α protein levels. When bEnd.3 cells were adapted to 18, 5 or 1 kPa O_2_, HIF-1α stabilization was only detected under hypoxia (Fig. 2C), confirming the absence of a hypoxic phenotype in cells maintained long-term under physiological normoxia (5 kPa O_2_).

### 3.4 Effects of ambient O_2_ levels on cell viability, ATP and GSH content and proliferation

Adaptation of bEnd.3 cells to 5 kPa O_2_ had no effect on cell viability, as evidenced by negligible changes in mitochondrial dehydrogenase activity (data not shown) or intracellular ATP levels (5 kPa O_2_: 24.3 ± 2.2 *vs* 18 kPa O_2_: 20.7 ± 1.7 nmol/mg.protein) (Fig. 2D). Intracellular GSH (Fig. 2E) and catalase (Fig. 2F) levels were significantly lower in bEnd.3 cells adapted to 5 kPa O_2,_ consistent with our previous findings in airway epithelial cells [23] and other studies in epidermoid carcinoma cells [40]. Total intracellular GSH levels were similar in bEnd.3 cells in passages 7–15 (data not shown). Moreover, bEnd.3 cell proliferation was decreased under 5 kPa O_2_ compared to 18 kPa O_2_ (Fig. 2G). The implications of these findings are that the enhanced oxidative stress during standard cell culture under hyperoxia (18 kPa O_2_) is attenuated in cells adapted to physiological normoxia 5 kPa O_2_).

### 3.5 Physiological normoxia attenuates sulforaphane induced Nrf2 regulated enzyme expression

**Fig. 3:**
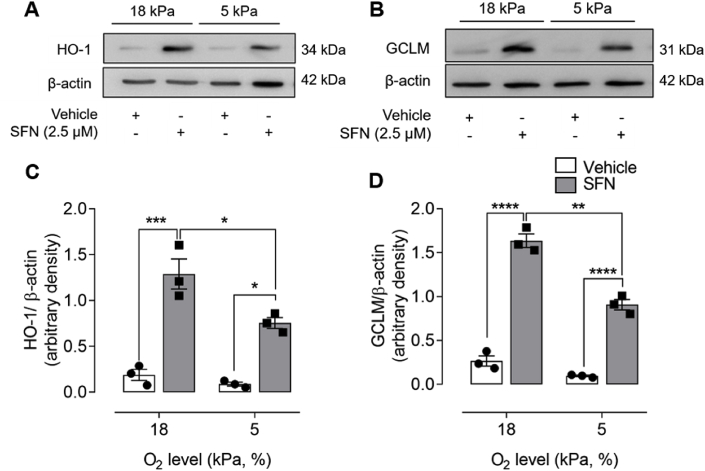
**Adaptation to physiological normoxia diminishes sulforaphane induced HO-1 and GCLM protein expression** bEnd 3 cells were cultured under either 18 or 5 kPa O_2_ for 5 d and then treated with vehicle (0.01% DMSO) or sulforaphane (SFN, 2.5 μM) for 24 h. Cell lysates were immunoblotted for HO-1 (**A**) and GCLM (**B**) expression relative to β-actin and analysed by densitometry (**C–D**). Data denote mean ± S.E.M., n = 3 independent bEnd.3 cultures, two-way ANOVA, *P < 0.05. **P < 0.01,***P < 0.001, ****P < 0.0001.

To determine whether Nrf2 redox signaling was affected by changes in ambient O_2_ levels, bEnd.3 cells were adapted to 18 or 5 kPa O_2_ and Nrf2 induced antioxidant enzyme expression determined by immunoblotting. Although basal levels of HO-1 and GCLM expression were affected negligibly following adaptation to physiological normoxia (5 kPa O_2_), upregulation of Nrf2 regulated enzyme expression by SFN (2.5 μM, 24 h) was significantly attenuated in cells adapted to 5 kPa O_2_ (Fig. 3). These findings are consistent with reports of diminished induction of antioxidant enzymes by Nrf2 in HUVEC [22], airway epithelial cells [23], RAW264.7 macrophages [44] and epidermoid carcinoma cells [40] under physiological normoxia.

### 3.6 Reoxygenation induced superoxide production in bEnd.3 cells under 18 kPa O_2_

**Fig. 4:**
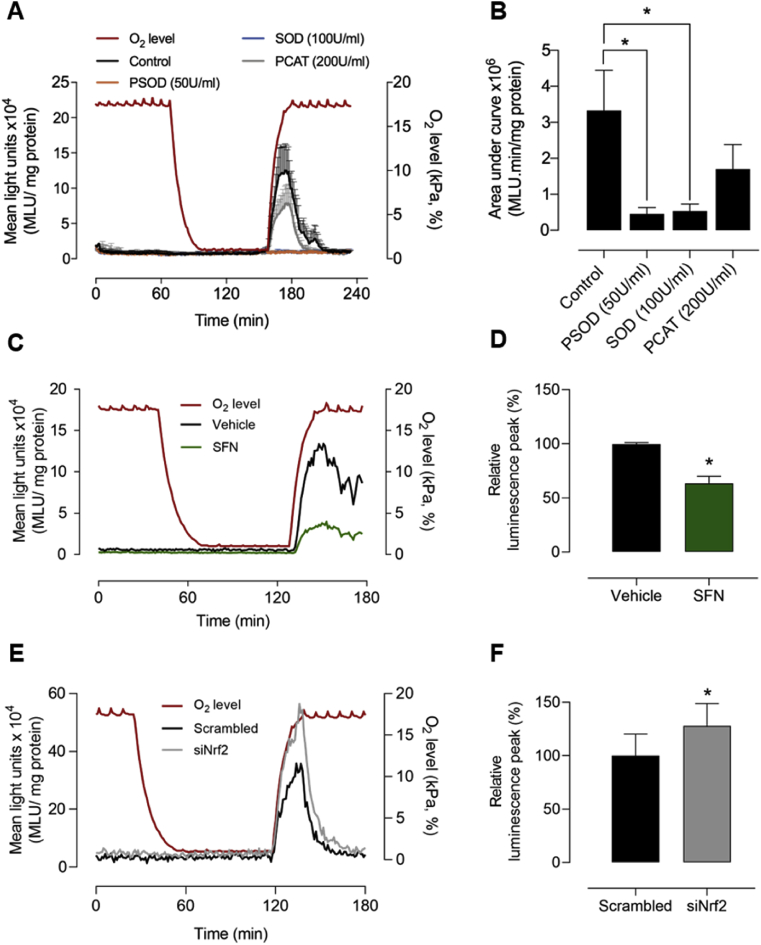
**Sulforaphane reduces and Nrf2 gene silencing enhances reoxygenation induced superoxide production in bEnd.3 cells under 18 kPa O**_**2**_ bEnd.3 cells were cultured under 18 kPa O_2_ for 5 d. (**A**) Cells were treated with vehicle (0.01% DMSO), PEG-superoxide dismutase (PSOD, 50U/ml), superoxide dismutase (SOD, 100U/ml) or PEG-catalase (PCAT, 200U/ml) for 30 min prior to incubation with L-012 (see Methods). Cells were rapidly transferred to an O_2_-regulated plate reader and subjected to hypoxia (1 h) and reoxygenation under 18 kPa O_2_, with L-012 luminescence measured in the absence (control) or presence of inhibitors. O_2_ levels inside the plate reader are indicated by the red line (right axis) and mean light units (MLU/mg protein) on the left axis. (**B**) Area under L-012 traces during reoxygenation (160–190 min) for each treatment. (**C**) Representative L-012 traces in cells pre-treated with vehicle (0.01% DMSO) or SFN (2.5 μM, 24 h) and (**D**) relative peak luminescence (120–140 min, % vehicle) on reoxygenation. (**E**) Representative L-012 traces in cells transfected with scrambled or Nrf2 siRNA and (**F**) relative peak luminescence (125–140 min, % scrambled siRNA). Data denote mean ± S.E.M., n = 3–4 independent bEnd.3 cultures, one-way ANOVA, *P < 0.05.

bEnd.3 cells adapted to 18 kPa O_2_ were incubated with the chemiluminescent probe L-012 to investigate reactive oxygen species generation during hypoxia-reoxygenation. As shown in Fig. 4A, reoxygenation induced free radical generation was significantly inhibited by SOD (100U/ml) and PEG-SOD (PSOD, 50U/ml), whereas PEG-CAT (PCAT, 200U/ml) led to a non-significant decrease in L-012 luminescence, suggesting that reoxygenation most likely increases superoxide generation. Fig. 4B summarizes the changes in luminescence induced by reoxygenation in the absence and presence of scavengers of reactive oxygen species.

As NADP(H) oxidases (NOX) have been implicated as a source of free radical generation in cerebral ischemia-reperfusion [45,46], bEnd.3 cells adapted to 18 kPa O_2_ were pre-treated with the NOX inhibitor VAS2870 (5 μM) for 30 min, incubated with L-012 and then exposed to hypoxia (1 kPa O_2_, 1 h) and reoxygenation. As shown in Supplementary Fig. S2, reoxygenation induced increases in L-012 luminescence were unaffected by VAS2870, suggesting that NADPH oxidases are an unlikely source of acute reoxygenation induced free radical generation in bEnd.3 cells.

### 3.7 Sulforaphane pretreatment protects against reoxygenation induced superoxide generation

To determine whether upregulation of Nrf2 target genes attenuates reoxygenation induced free radical generation, bEnd.3 cells were adapted to 18 kPa O_2_ and pre-treated with either vehicle (DMSO 0.01%) or SFN (2.5 μM) for 24 h. SFN significantly diminished reoxygenation induced L-012 luminescence (Fig. 4C and D) and moreover, in cells transfected with scrambled or Nrf2 siRNA, we confirmed that silencing Nrf2 significantly enhances the L-012 luminescence signal during reoxygenation (Fig. 4E and F).

### 3.8 Reoxygenation induced free radical generation is diminished in bEnd.3 cells under 5 kPa O_2_

**Fig. 5:**
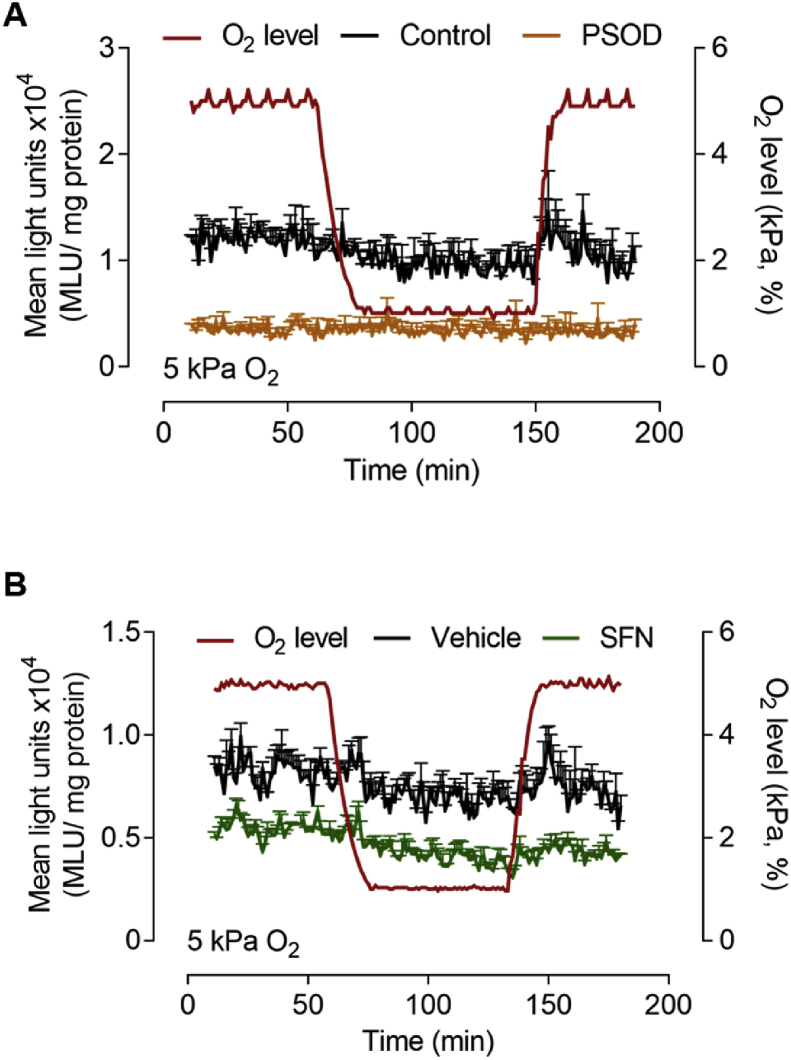
**Adaptation to 5 kPa O**_**2**_**diminishes reoxygenation induced reactive oxygen species generation in bEnd.3 cells** bEnd.3 cells were cultured under 5 kPa O_2_ for 5 d. (**A**) Cells were treated with vehicle (0.01% DMSO, black line, control) or PEG-superoxide dismutase (PSOD, 50 U/ml, orange line) for 30 min prior to incubation with L-012. Cells were transferred to an O_2_-regulated plate reader and subjected to hypoxia (1 h) and reoxygenation under 5 kPa O_2_. O_2_ levels inside the plate reader are indicated by the red line (right axis) and mean light units (MLU/mg protein) on the left axis. (**B**) bEnd.3 cells pre-treated with vehicle (0.01% DMSO) or SFN (2.5 μM, 24 h) prior to hypoxia (1 h) and reoxygenation under 5 kPa O_2_. Data denote mean ± S.E.M, n = 3 independent bEnd.3 cultures. (For interpretation of the references to color in this figure legend, the reader is referred to the Web version of this article.)

Basal and reoxygenation induced L-012 luminescence was significantly lower in bEnd.3 cells adapted to 5 kPa O_2_ (Fig. 5A) compared to 18 kPa O_2_ (Fig. 4A). Although reoxygenation induced changes in L-012 luminescence were not significant, the signal appeared decreased in the presence of PSOD (Fig. 5A) or following pretreatment of cells with SFN (2.5 μM, 24 h) (Fig. 5A and B). This attenuated intracellular free radical production in bEnd.3 cells under 5 kPa O_2_ is consistent with previous studies in RAW264.7 macrophages [44], epidermoid carcinoma cells [40], SH-SY5Y neuronal cells [47] and skeletal myoblasts/myotubes [48].

### 3.9 Reoxygenation-induced increases in MitoSOX red fluorescence

**Fig. 6:**
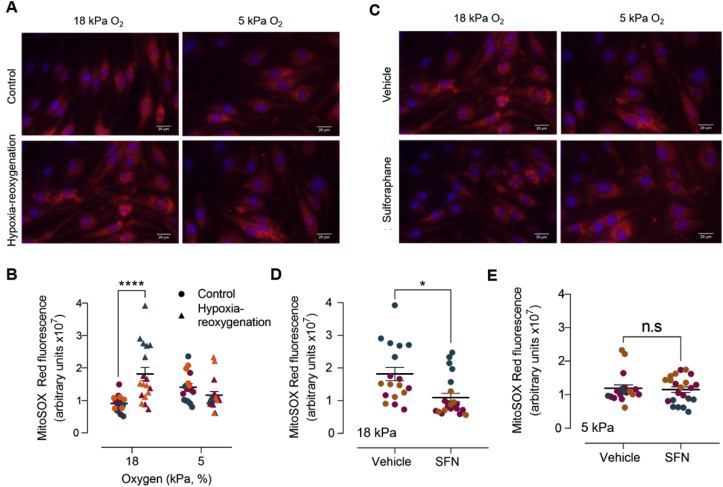
**Effects of sulforaphane pretreatment on reoxygenation induced mitochondrial reactive oxygen species generation in bEnd.3 cells adapted to 18 kPa or 5 kPa O**_**2**_ bEnd.3 cells seeded in Ibidi μ-Slide 8-well chambers were cultured under 18 or 5 kPa O_2_ for 5 d. Cells were subjected to hypoxia (1 kPa O_2_, 1 h) and loaded with MitoSOX™ Red for 5 min before the start of 30 min reoxygenation under 18 or 5 kPa O_2_, respectively. Control cells were loaded with MitoSOX™ Red during the last 30 min of an experiment. Cells were fixed with 4% paraformaldehyde and images acquired using a Nikon Diaphot microscope with a 40× objective. (**A**) Representative images of MitoSOX fluorescence and DAPI stained nuclei after 30 min reoxygenation and (**B**) quantification of MitoSOX fluorescence. (**C**) bEnd.3 cells were pre-treated with vehicle (0.01% DMSO) or SFN (2.5 μM) for 24 h before exposure to hypoxia (1 h) and reoxygenation under 18 or 5 kPa O_2_, respectively. Representative images of MitoSOX fluorescence and DAPI stained nuclei after 30 min reoxygenation and (**D–E**) quantitation of MitoSOX fluorescence. Each symbol in panels B, D and E represents the mean fluorescence from at least 10 cells in a field of view, with each color denoting a different bEnd.3 experiment with at least 6 different fields of view. Data denote mean ± S.E.M., n = 18–20 fields of view in each of 3 independent bEnd.3 cell cultures, two-way ANOVA followed by Bonferroni post-hoc analysis, ****P < 0.0001. Scale bar = 20 μm. (For interpretation of the references to color in this figure legend, the reader is referred to the Web version of this article.)

To further validate reoxygenation mediated changes in L-012 luminescence, we examined mitochondrial reactive oxygen species generation in bEnd.3 cells adapted to 18 or 5 kPa O_2_. Hypoxia-reoxygenation increased MitoSOX fluorescence in cells under 18 kPa O_2_ with negligible changes detectable under 5 kPa O_2_ (Fig. 6A and B). As shown in Supplementary Fig. S3, reoxygenation induced increases in MitoSOX fluorescence were unaffected by inhibition of complex I (rotenone, 1 μM) or eNOS (l-NAME, 100 μM). Notably, pretreatment of cells with SFN (2.5 μM, 12 h) significantly attenuated reoxygenation induced MitoSOX fluorescence in cells under 18 but not 5 kPa O_2_ (Fig. 6C–E), consistent with the negligible changes in L-012 luminescence observed in bEnd.3 cells during acute reoxygenation under 5 kPa. To exclude the possibility that changes in ambient O_2_ levels affected MitoSOX fluorescence, the probe was dissolved in 1% FCS medium and a 96-well plate transferred to the plate reader under 18 or 5 kPa O_2_. When H_2_O_2_ (10 μM) was injected using on-board injector units in the plate reader, MitoSOX fluorescence was similar under 18 and 5 kPa O_2_ (data not shown).

### 3.10 Reoxygenation-induced increases in FeRhoNox fluorescence

**Fig. 7:**
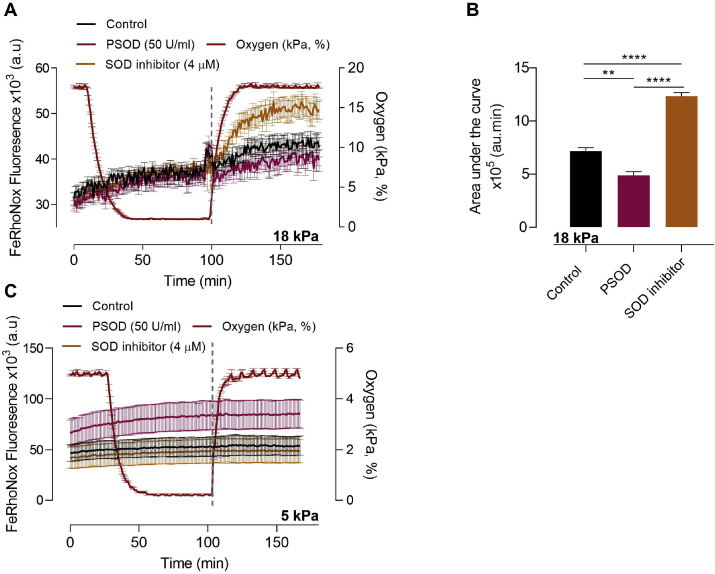
**Reoxygenation induces intracellular Fe**^**2+**^**release in bEnd.3 cells adapted to 18 kPa O**_**2**_**but not 5 kPa O**_**2**_. bEnd.3 cells were cultured under 18 or 5 kPa O_2_ for 5 d. Cells were then incubated with the Fe^2+^-selective probe FeRhoNox™-1 (5 μM, 1 h) in HBSS in the presence of vehicle (0.01% DMSO, control, black line), PEG-superoxide dismutase (PSOD, 50U/ml, pink line) or a SOD inhibitor (4 μM ammonium tetrathiomolybdate, orange line). (**A-B**) Mean FeRhoNox fluorescence traces in cells exposed to hyperoxia (18 kPa O_2_), hypoxia (1 kPa O_2_, 1 h) and reoxygenation under 18 kPa O_2_ and area under the curve following reoxygenation (dashed line indicates reoxyenation period 100–160 min) for each treatment. (**C**) Mean FeRhoNox fluorescence traces in cells adapted to physiological normoxia (5 kPa O_2_), hypoxia (1 kPa O_2_, 1 h) and reoxygenation under 5 kPa O_2_. Data denote mean ± S.E.M from 3 to 4 independent bEnd.3 cell cultures, one-way ANOVA followed by Bonferroni post-hoc analysis, **P < 0.001, ****P < 0.0001. (For interpretation of the references to color in this figure legend, the reader is referred to the Web version of this article.)

Based on caveats associated with the specificity of L-012 and MitoSOX Red, such as non-specific oxidation of the probes [49,50], further indirect measurements of reoxygenation induced superoxide production were conducted using an Fe^2+^-specific fluorescent indicator, FeRhoNox™-1, in bEnd.3 adapted to 18 or 5 kPa O_2_. Inhibition of superoxide dismutase by ammonium tetrathiomolybdate has been reported to prolong the cytosolic Fe^2+^ signal in dermal fibroblasts and endothelial cells challenged with UVA radiation [37]. As shown in Fig. 7A, increases in FeRhoNox-1 fluorescence during reoxygenation under 18 kPa O_2_ were inhibited by PEG-SOD and potentiated by inhibition of SOD with ammonium tetrathiomolybdate. Previous studies have shown that mitochondria exposed to superoxide release iron from iron-sulphur clusters, indicating that increased radical production will lead to an increase in free iron [51,52]. In the present study, we exploited the fact that increases in free iron would increase FeRhoNox™-1 fluorescence, and thus changes in fluorescence served as an indirect measure of superoxide generation in bEnd.3 cells subjected to reoxygenation. Notably, reoxygenation induced FeRhoNox-1 fluorescence was attenuated in bEnd.3 cells adapted to 5 kPa O_2_ (Fig. 7B). Together with our findings of reoxygenation induced changes in L-012 luminescence and MitoSOX fluorescence, FeRhoNox-1 measurements suggest that superoxide is the most likely free radical generated during acute reoxygenation induced oxidative stress in bEnd.3 cells.

## 4 Discussion

Changes in ambient O_2_ levels during cell culture *in vitro* alter (i) ion channel and kinase activities [[53], [54], [55]], (ii) endothelial Ca^2+^ signaling, nitric oxide bioavailability and their sensitivity to Ca^2+^ overload [24,25], and (iii) induction of Nrf2-targeted antioxidant defenses [22,23,56]. We here further demonstrate that the redox phenotype of mouse brain microvascular endothelial cells is critically affected by ambient oxygen levels. Endothelial cells lining the blood-brain barrier *in vivo* are exposed to O_2_ levels ranging between ~3 and 7 kPa, yet the majority of studies in brain endothelial and other cell types *in vitro* have employed standard culture conditions in which cells are exposed to hyperoxia (18 kPa O_2_) and therefore sustained oxidative stress [20].

Using the O_2_-sensitive nanoparticle probe MitoXpress®-INTRA, we obtained the first measurements of intracellular O_2_ (3.4 kPa) in bEnd.3 endothelial cells, recapitulating O_2_ levels measured in brain endothelium *in vivo*. Importantly, long-term adaptation of bEnd.3 cells to 5 kPa O_2_ was not associated with HIF-1α stabilization, confirming the absence of a hypoxic phenotype under physiological normoxia. Moreover, as gradients exist between ambient O_2_ levels in a Scitive workstation, medium and cytosol, it is critical that medium and intracellular O_2_ levels are measured simultaneously [20,22]. MitoXpress®-INTRA has been used to measure intracellular O_2_ in umbilical vein endothelial cells [22,24], mouse embryonic fibroblasts [26], cortical neurons [57] and now in brain microvascular endothelial cells.

Adaptation of bEnd.3 cells under 5 kPa O_2_ did not affect cell viability or intracellular ATP levels, but significantly decreased levels of intracellular GSH and catalase, suggesting that cells under physiological normoxia experience less oxidative stress [20,58]. Basal expression of Nrf2-regulated antioxidant enzymes was similar in bEnd.3 cells cultured under 18 or 5 kPa O_2_, however SFN mediated induction of HO-1 and GCLM was significantly attenuated in cells adapted to 5 kPa O_2_. Our finding of diminished HO-1 induction is in agreement with our previous studies in human umbilical vein and coronary artery endothelial cells [22] and other studies in lung epithelial cells [23] and human dental pulp stem cells [56] cultured under relevant physiological O_2_ levels. Notably, electrophile and nitric oxide mediated induction of HO-1 in human endothelial cells adapted 5 kPa O_2_ is attenuated, but reversible on re-exposure of cells to 18 kPa O_2_ or following silencing of the Nrf2 repressor Bach1 [22].

Our previous studies of reperfusion injury in a rodent model of transient ischemic stroke established that activation of Nrf2 antioxidant defenses by SFN affords neurovascular and neurological protection [17,18]. To mimic ischemia-reperfusion injury in stroke at a cellular level, bEnd.3 cells were adapted to either 18 or 5 kPa O_2_ and subjected to hypoxia (1 kPa O_2_) and reoxygenation under 18 or 5 kPa O_2_, respectively. Reoxygenation-induced increases in L-012 luminescence in cells adapted to 18 kPa O_2_ was abrogated by SOD and polyethylene glycol SOD, implicating superoxide as the most likely free radical species generated during reoxygenation. Although polyethylene glycol catalase led to a non-significant decrease in reoxygenation-induced free radical production, we cannot exclude that inhibition of L-012 luminescence signal by SOD or polyethylene glycol SOD may be due to generation of superoxide from molecular oxygen during L-012 oxidation by H_2_O_2_/peroxidase [59]. We further demonstrated that upregulation of Nrf2-regulated antioxidant enzymes by SFN led to a significant decrease in reoxygenation induced free radicals (Fig. 4C and D), whilst silencing Nrf2 transcriptional activity enhanced reoxygenation induced free radical generation (Fig. 4E and F).

Reoxygenation induced changes in L-012 luminescence were lower in bEnd.3 cells adapted to 5 kPa O_2_ (Fig. 5), although L-012 signals trended to decrease in the presence of PEG-SOD or following SFN pretreatment. In this context, studies in macrophages [44], epidermoid carcinoma cells [40] and dental pulp stem cells [60], as well as, our experiments in human endothelial cells (data not shown) confirm that oxidative stress is lower in cells adapted to physiological normoxia. Thus, under standard, hyperoxic cell culture conditions, the redox phenotype of cells is characterized by an upregulation of Nrf2-regulated gene transcription to counteract enhanced reactive oxygen species generation and sustained oxidative stress [20].

We further investigated the redox status of bEnd.3 cells exposed to hypoxia-reoxygenation by assaying MitoSOX fluorescence as an index of mitochondrial reactive oxygen species generation. Reoxygenation significantly increased MitoSOX fluorescence in bEnd.3 cells adapted to 18 but not 5 kPa O_2_, and notably SFN pretreatment only attenuated reoxygenation-induced MitoSOX fluorescence in cells adapted to 18 kPa O_2_, further supporting our finding that SFN inhibits acute reoxygenation induced increases in L-012 luminescence. Reoxygenation induced increases MitoSOX fluorescence were unaffected by l-NAME or a pan-NADPH oxidase inhibitor (VAS2870), suggesting that free radical generation in bEnd.3 cells was unlikely due to eNOS or NOX. Although VAS2870 had no effect on cell viability or reoxygenation induced free radical generation, we cannot exclude that VAS2870 and other NOX inhibitors may have off-target effects via thiol alkylation, inhibition of mitochondrial respiration and cytotoxicity [61]. Furthermore, although undetectable in our study, we cannot exclude the possibility of enhanced reactive oxygen species generation during hypoxia, as it has recently been suggested that acute hypoxia drives the import of Na^+^ into the mitochondrial matrix, reducing inner mitochondrial fluidity and consequently concentrating the production of superoxide at complex III [62].

To further characterize reoxygenation-induced free radical generation in bEnd.3 cells, release of intracellular Fe^2+^ was monitored as an indirect measure of intracellular superoxide generation. By using PEG-SOD and a SOD inhibitor, we demonstrated for the first time that changes in FeRhoNox-1 fluorescence provide a useful measure of reoxygenation-induced superoxide generation in brain microvascular endothelial cells. Release of labile iron is closely associated with reactive oxygen species generation, and iron accumulation occurs in stroke [63], traumatic brain injury [64] and neurodegenerative disorders [65,66]. Furthermore, mitochondria exposed to superoxide anions release iron from iron-sulphur clusters, such that increased free radical generation will result in increased free iron [51,52]. Increases in superoxide in the presence of SOD inhibition reduces Fe^3+^ in the ferritin core to Fe^2+^, releasing Fe^2+^ into the cytoplasm [37,67].

Our study establishes that bEnd.3 cells adapted to long-term to hyperoxia (18 kPa O_2_) exhibit heightened sensitivity to hypoxia-reoxygenation, resulting in increased reactive oxygen species generation on reoxygenation. Studies *in vivo* have reported that following ischemia-reperfusion injury in the heart and brain, accumulation of succinate in mitochondria drives reactive oxygen species generation via reverse electron transport at mitochondrial complex I, and that oxidative damage can be decreased by reducing succinate accumulation [68]. In the present study, activation of Nrf2 by SFN significantly diminished reoxygenation induced free radical generation while silencing of Nrf2 exacerbated free radical generation, implicating Nrf2 in protection against reoxygenation/reperfusion injury. In this context, Nrf2 has been shown to significantly affect the mitochondrial membrane potential, fatty acid oxidation and the availability of substrates including succinate [69,70]. As Nrf2 deficient cells and mice *in vivo* are more sensitive to oxidative damage [71,72], activation of Nrf2 by SFN not only upregulates antioxidant defense enzymes but importantly also influences mitochondrial substrate utilization and respiration [69,70].

As generation of reactive oxygen species was attenuated in bEnd.3 cells adapted to physiological normoxia, it is possible that the probes L-012 and MitoSOX Red used in this study and other studies lack sufficient sensitivity to monitor low levels of radical generation in response to acute reoxygenation. Although recent advances in multiphoton redox and pO_2_ imaging have enabled elegant quantification of metabolic processes under different ambient O_2_ levels [73], we consider it important to ensure that decreasing ambient oxygen levels from 18 kPa O_2_ does not result in HIF-1α stabilization and activation hypoxic signaling pathways. In this context, we previously reported that long-term (~5 d) culture of vascular cells under physiological O_2_ levels is required to exclude a hypoxic phenotype [22,24].

In view of the caveats concerning luminescence and fluorescence indicators [49,50], further studies are warranted using novel genetic biosensors for high-resolution, real-time imaging of reactive oxygen and nitrogen species in single cells and subcellular compartments [74,75]. Conducting such experiments in cells adapted long-term under controlled and physiologically relevant O_2_ levels will prove challenging, but we are convinced that such *in vitro* cell culture models, in particular targeting biosensors to mitochondria in live cells, will provide insights for the design of novel therapeutics for treatment cerebral, coronary, renal and hepatic ischemia-perfusion injury.

## Author contributions

G.W., P.A.F. and G.E.M. conceptualized the study; G.W. developed the methodology, T.P.K. assisted with MitoXpress®-Intra experiments and R.C.M.S. with FeRhoNox-1 experiments; G.W., S.S. and M.J.S. performed the experiments; G.W. and G.E.M. wrote the manuscript which was reviewed by all authors. G.E.M. is the guarantor of this study, with responsibility for the integrity of the data and accuracy of the data analysis.

## Declaration of competing interest

The authors declare no competing interests.
