# Supplementary material for: Nrf2-regulated redox signaling in brain endothelial cells adapted to physiological oxygen levels: Consequences for sulforaphane mediated protection against hypoxia-reoxygenation
Source: Redox Biol. 2020 Sep 8;37:101708. doi: 10.1016/j.redox.2020.101708 (PMC7502377; doi:10.1016/j.redox.2020.101708)
Supplement: Multimedia component 1 [file mmc1.docx]

**Appendix A. Supplementary data**

**Table S1. Primers for qPCR**

| **Target gene** | **Primer sequence** |
| --- | --- |
| HO-1 | Forward: CAACATTGAGCTGTTTGAGGAG  Reverse: CTCTGACGAAGTGACGCCAT |
| NQO1 | Forward: CCTTTCCAGAATAAGAAGACCTTGC  Reverse: GAAGCCACAGAAACGCAGGA |
| Bach1 | Forward: CGGAAA TCGAGAAGCTGCAAAG  Reverse: AAAAGGAAAGCGGGCAGTCG |
| Keap1 | Forward: ATCTACGCAGTCGGGGGTTC  Reverse: CCCGCTCTGGCTCATATCTCTC |
| SDHA | Forward: CAAAAACAGACCTGCGGCTT  Reverse: CTGGGTATTGAGTAGAAATTGCATC |
| B2M | Forward: GTCGCTTCAGTCGTCAGCA  Reverse: TTGAGGGGTTTTCTGGATAGCA |
| RPL13A | Forward: GAGGGGGCAGGTTCTGGTATT  Reverse: CGGGAGGGGTTGGTATTCAT |
|  |  |

*Abbreviations*: HO-1, heme oxygenase; NQO1, NADPH quinone oxidoreductase 1; Bach1, BTB and CNC homology 1; Keap1, kelch-like-ECH-associated protein 1; SDHA, succinate dehydrogenase complex flavoprotein subunit A; B2M, beta-2-microglobulin; RPL13A, ribosomal protein L13A.


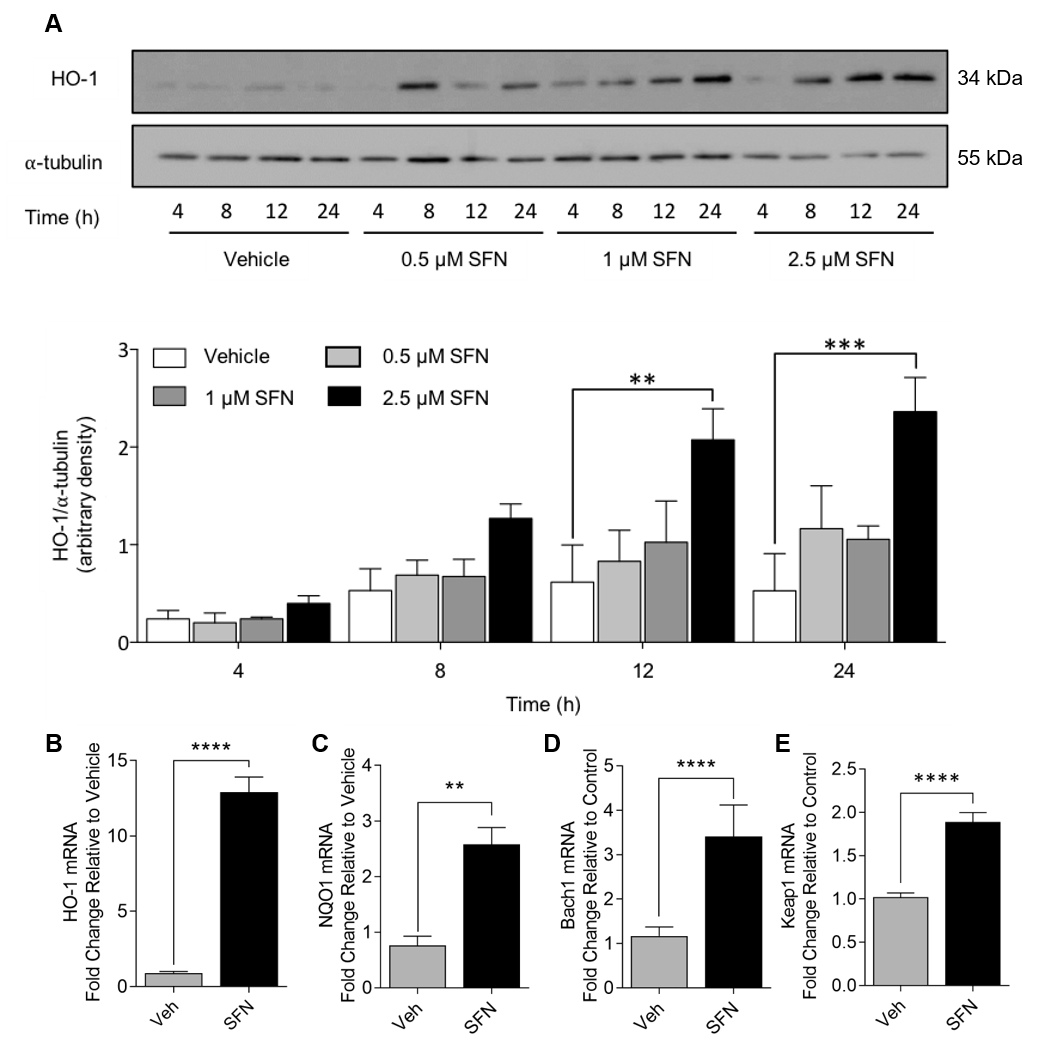


Fig. S1. Time- and concentration-dependent effects of sulforaphane on Nrf2-regulated protein and gene expression in bEnd.3 endothelial cells

(A) bEnd.3 cells were treated for 4, 8, 12 and 24 h with vehicle (0.01% DMSO) or sulforaphane (SFN, 0.5–2.5μM) and HO-1 protein expression determined relative to α-tubulin and analysed densitometrically. Data denote mean ± S.E.M., n = 4 independent cell cultures, two-way ANOVA with Tukeys post-test, **P<0.01, ***P<0.001. (B-E) bEnd.3 cells treated with vehicle (0.01% DMSO) or sulforaphane (SFN, 2.5μM) for 4 h and mRNA expression of HO-1 (B), NQO1 (C), Bach1 (D) and Keap1(E) determined by qPCR and normalized to three housekeeping genes (SDHA, RPL13A and B2M). Data denote mean ± S.E.M., n = 5 independent cell cultures, Student’s *t*-test, ** P<0.01, ****P<0.0001.


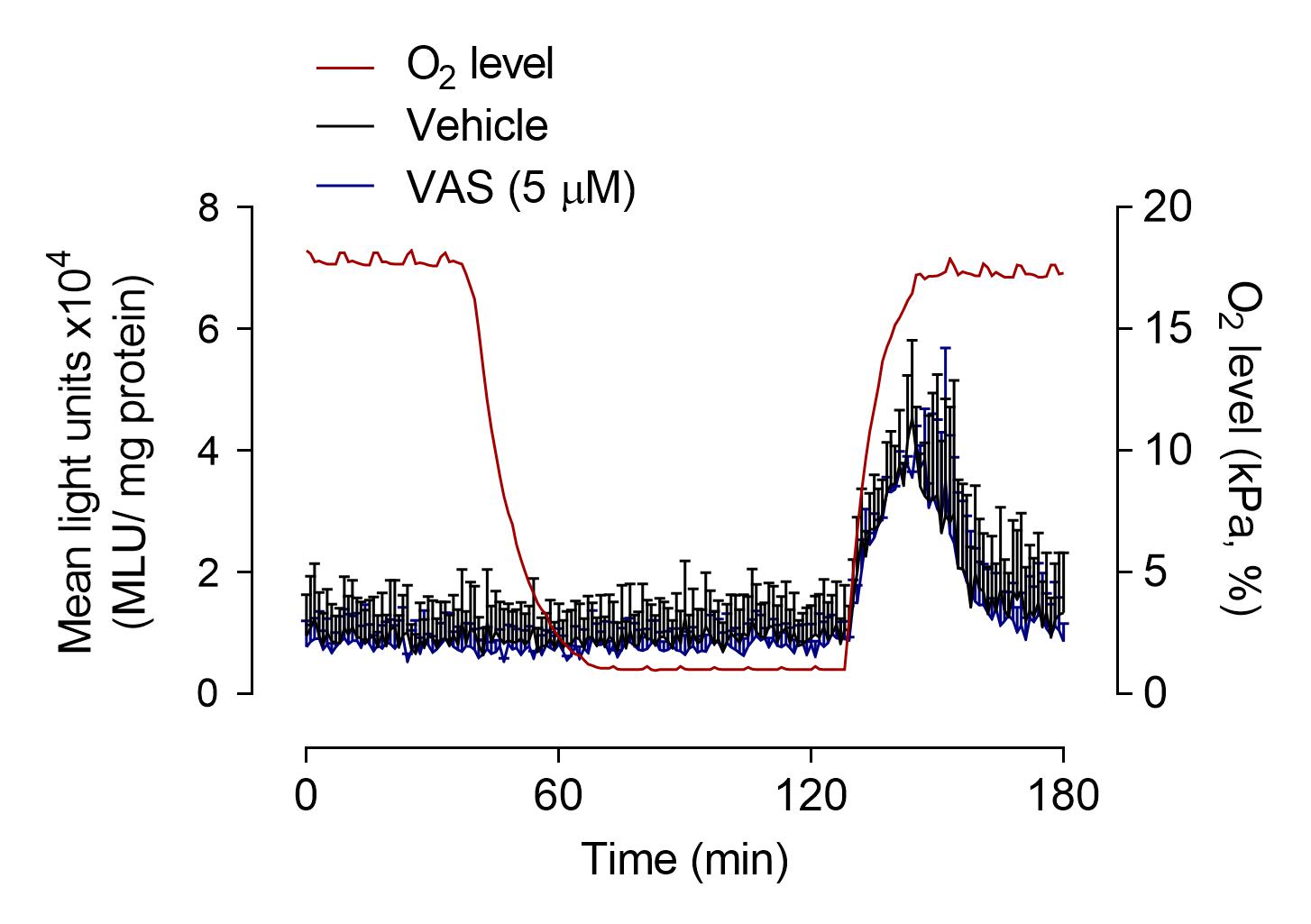


**Fig. S2. NADPH oxidase inhibitor VAS2870 does not inhibit reoxygenation induced L-012 luminescence**

bEnd.3 cells were cultured under 18 kPa O_2_ for 5 d. Cells were treated with vehicle (vehicle, 0.01% DMSO; black line) or the NOX inhibitor VAS2870 [31] (VAS, 5μM; blue line) for 30 min prior to incubation with L-012 (see Methods). Cells were then transferred to an O_2_-regulated plate reader and subjected to hypoxia (1 h) and reoxygenation under 18 kPa O_2_ and L-012 luminescence measured in the absence or presence of VAS2870. O_2_ levels inside the plate reader are indicated by the red line (right axis) and mean light units (MLU/mg protein) on the left axis. Data denote mean ± S.E.M from 3 independent bEnd.3 cell cultures.


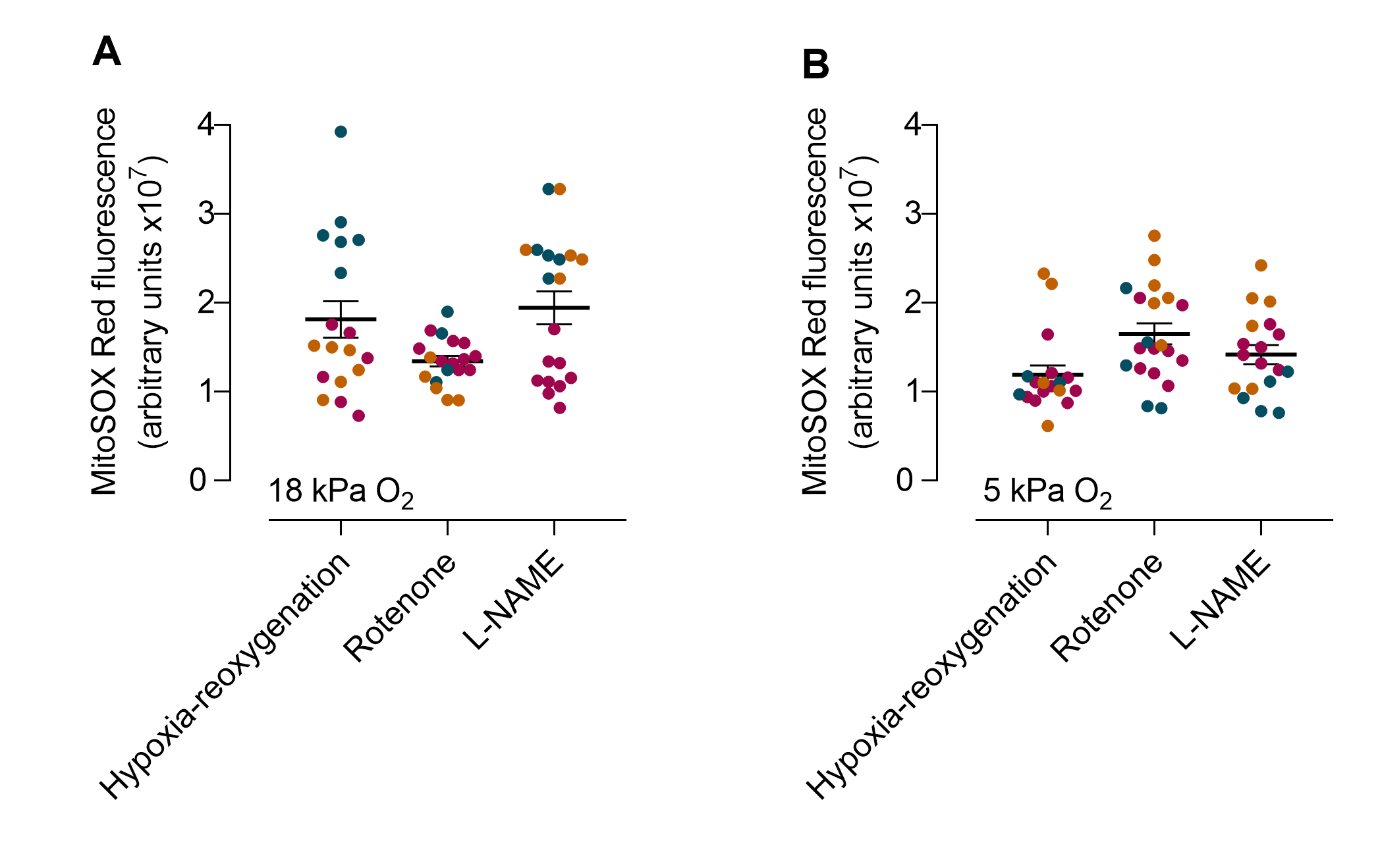


**Fig. S3. Effects of rotenone and L-NAME on MitoSOX Red fluorescence during reoxygenation of bEnd.3 cells under 18 kPa or 5 kPa O_2_**.

bEnd.3 cells were cultured under 18 kPa O_2_ (A) or 5 kPa O_2_ (B) for 5 d and then subjected to hypoxia (1 kPa O_2_, 1 h) and reoxygenation under 18 or 5 kPa O_2_, respectively. Cells were loaded with MitoSOX™ Red for 5 min before the start of 30 min reoxygenation in the absence (*hypoxia-reoxygenation in absence of inhibitors*) or presence of rotenone (1μM, complex 1 inhibitor) or L-NAME (100μM, eNOS inhibitor). Cells were fixed with 4% paraformaldehyde and images acquired using a Nikon Diaphot microscope with a 40x objective and fluorescence quantified using ImageJ. Each symbol represents the mean fluorescence from at least 10 cells in a field of view, with each color denoting a different bEnd.3 experiment with at least 6 different fields of view. Data denote mean ± S.E.M., n = 18 – 20 fields of view in each of 3 independent bEnd.3 cell cultures (measurements obtained in the same bEnd.3 cell cultures depicted in Fig. 6).
